# ErbB2 and bone sialoprotein as markers for metastatic osteosarcoma cells

**DOI:** 10.1038/sj.bjc.6600735

**Published:** 2003-02-10

**Authors:** G Valabrega, F Fagioli, S Corso, E Madon, A Brach del Prever, E Biasin, A Linari, M Aglietta, S Giordano

**Affiliations:** 1 Institute for Cancer Research and Treatment (IRCC), University of Torino Medical School, Str. Prov. 142, Km 3.95, 10060 Candiolo, Italy; 2 Department of Pediatrics, University of Torino, Torino, Italy; 3 Department of Pathology, Regina Margherita Children's Hospital, Torino, Italy

**Keywords:** osteosarcoma, apheresis, ErbB2, bone sialoprotein, tumour marker

## Abstract

Osteosarcoma is the most common malignant bone neoplasia occurring in young patients in the first two decades of life, and represents 20% of all primitive malignant bone tumours. At present, treatment of metastatic osteosarcoma is unsatisfactory. High-dose chemotherapy followed by CD34+ leukapheresis rescue may improve these poor results. Neoplastic cells contaminating the apheresis may, however, contribute to relapse. To identify markers suitable for detecting osteosarcoma cells in aphereses we analysed the expression of bone-specific genes (Bone Sialoprotein (BSP) and Osteocalcin) and oncogenes (Met and ErbB2) in 22 patients with metastatic osteosarcoma and six healthy stem cell donors. The expression of these genes in aphereses of patients affected by metastatic osteosarcoma was assessed by RT–PCR and Southern blot analysis. Met and Osteocalcin proved to be not useful markers since they are positive in aphereses of both patients with metastatic osteosarcoma and healthy stem cell donors. On the contrary, BSP was expressed at significant levels in 85% of patients. Moreover, 18% of patients showed a strong and significantly positive (seven to 16 times higher than healthy stem cell donors) ErbB2 expression. In all positive cases, neoplastic tissue also expressed ErbB2. Our data show that ErbB2 can be a useful marker for tumour contamination in aphereses of patients affected by ErbB2-expressing osteosarcomas and that analysis of Bone Sialoprotein expression can be an alternative useful marker.

Osteosarcoma, the most common malignant bone neoplasia occurring in the first two decades of life, is frequently associated with alterations of Rb (Benedict *et al*, 1988), p53 (Varley *et al*, 1997), mdm2 (Wunder *et al*, 1999), c-myc (Gamberi *et al*, 1998), c-fos (Wu *et al*, 1990) and other genes. However, aetiology and molecular mechanisms underlying osteosarcoma remain unclear. Metastasis, an early event in high-grade osteosarcoma history, is the major cause of morbidity and mortality. At present, treatment of high-grade metastatic osteosarcoma is unsatisfactory as 5 years overall survival is only 20–25%. Increase of dose intensity of chemotherapeutic agents (high-dose chemotherapy) may improve these poor results, but it is associated with an increase of haematological and systemic toxicity (Santana *et al*, 1992). Recent use of haemopoietic growth factors has allowed, on one hand, the reduction of interval and intensity of neutropenia and, on the other, the possibility to harvest an adequate number of mobilized stem cells that, reinfused after myeloablative treatment, can induce a faster haemopoietic recovery (Fagioli *et al*, 2000). Reinfusion of stem cells has, however, generated concern about the possibility of contamination by neoplastic cells rescued from aphereses. Although it is not yet known whether relapse is because of endogenous reinfused cells, it is certainly important to investigate tumour contamination in leukaphereses.

Our aim was to find markers for detecting osteoblastic osteosarcoma cells in leukaphereses of patients with metastatic osteosarcoma. Unfortunately, neither specific translocation nor tumour-associated antigens have yet been described for this tumour. We therefore tried to assess the circulating tumour burden in patients with osteosarcoma metastatic at diagnosis. This was carried out by evaluating the expression of two kinds of molecules on cells derived from apheretic procedures: (a) Bone Sialoprotein (BSP) and Osteocalcin that are produced by bone cells, histotypically similar to osteosarcoma cells; (b) oncogenes like MET or ErbB2, frequently overexpressed in osteosarcomas.

Bone Sialoprotein is a 80 kDa extracellular matrix protein, mainly produced and secreted by osteoblasts (Bianco *et al*, 1991). BSP overexpression has been demonstrated also in breast (Bellahcene *et al*, 1994) and in prostate cancer (Waltregny *et al*, 1998) in which BSP overexpression is strongly associated with poor prognosis. Osteocalcin is a 5.8 kDa (Thiede *et al*, 1994) extracellular matrix bone protein of still unknown function, selectively produced by mature osteoblasts. Met is the transmembrane tyrosine kinase receptor for hepatocyte growth factor (HGF). It is overexpressed in 60% of osteosarcomas (Ferracini *et al*, 1995) and in many other tumours such as colon (Di Renzo *et al*, 1995), gastric (Carneiro and Sobrinho-Simoes, 2000; Lee *et al*, 2000) and renal (Schmidt *et al*, 1997) carcinomas. ErbB2 is a 185 kDa tyrosine kinase receptor of the EGF receptor family (Samanta *et al*, 1994). ErbB2 overexpression has been demonstrated in 30% of osteosarcomas (Gorlick *et al*, 1999) and in many other tumours such as breast (Pegram and Slamon, 2000), ovary (Slamon *et al*, 1989), lung (Scheurle *et al*, 2000) and gastric (Lin *et al*, 2000) carcinomas. Expression of ErbB2 in localised and metastatic osteosarcomas has been associated with poor prognosis in terms of survival, development of metastasis and response to chemotherapy (Onda *et al*, 1996; Pegram and Slamon, 2000).

As all the analysed markers are illegitimately expressed in haemopoietic cells (Chelly *et al*, 1989), we considered a semiquantitative approach using RT–PCR followed by Southern blot analysis, with a final quantification of radioactive intensity.

In this work, we show that Met and Osteocalcin are not useful markers for the detection of micrometastasis in aphereses of patients with metastatic osteosarcoma, as they are expressed at significant levels also in healthy donors. On the contrary, we show that BSP expression is significantly increased in patients. We then show that cells derived from apheretic procedures express ErbB2 at significant levels in patients with metastatic osteosarcomas in comparison with healthy stem cell donors. Finally, we show that different aphereses from the same patient display diverse ErbB2 levels.

## MATERIALS AND METHODS

### Patients and healthy donors

All patients and healthy stem cell donors gave written informed consent for leukapheretic procedures.

Experiments on Osteocalcin and BSP were carried out using leukaphereses from 14 patients. Expression of ErbB2 was evaluated on the same leukaphereses plus a further eight samples from patients successively recruited. All patients had histologically confirmed metastatic osteosarcoma, before apheresis collection (median age 12.8 years, range 6–34 years). Sites of primary osteosarcoma were femur, humerus, tibia and fibula (see Table 1

**Table 1:** Patient characteristics

Sex	
Females	9
Males	13
Age	
Median	12 years, 8 months
Range	6 years, 2 months–34 years, 10 months
Histology	
Osteoblastic	13
Fibroblastic	4
Chondroblastic	3
Teleangectasic	1
Chondro-osteoblastic	1
Site at diagnosis	
Humerus	3
Fibula	5
Femur	8
Tibia	5
Fibula+tibia	1
Site of MTS	
Lung	21
Lung+pelvis	1

). All patients had been treated with chemotherapy (following methodology described in Fagioli *et al*, 2002) prior to apheresis collection. As no primary tumours were available, ErbB2 expression was evaluated on lung metastases excised from patients undergoing surgery. Six aphereses from healthy stem cell donors were used as control. Healthy stem cell donors were siblings of patients with acute lymphoblastic leukaemia undergoing allogenic transplantation. Both osteosarcoma patients and healthy stem cell donors were treated with granulocyte colony-stimulating factor (G-CSF) before stem cell harvesting.

### RNA extraction, RT–PCR and Southern blot experiments

Mononucleated cells were isolated by Ficoll from 1 ml of leukapheresis from individuals treated with G-CSF, obtaining a number of cells included between 10^7^ and 2×10^7^. RNAs were purified with the ULTRASPEC II RNA kit (Biotecx Houston, TE, USA) based on extraction with guanidine and urea salts. DNA and proteins were separated by phenol–chloroform, then RNA was precipitated with ethanol 75%. RNA was finally eluted in DEPc (diethylpyrocarbonate) containing water. A measure of 1 *μ*g of RNA was retro transcribed using the Promega kit (Madison, WI, USA). In all, 100 ng of cDNA was used for RT–PCR experiments.


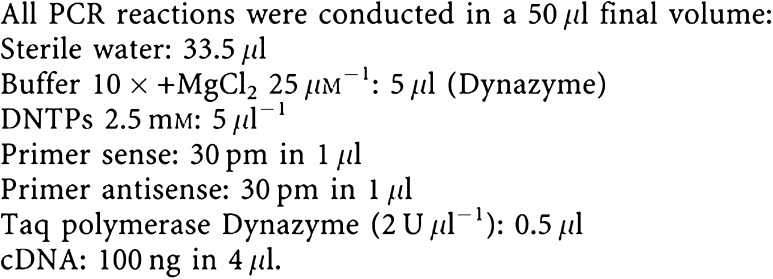


To quantify cDNA and to verify the absence of degradation, we performed PCR amplification of mRNAs coding either for GAPDH or *β*-actin, ubiquitously expressed proteins. All PCR experiments were preceded by a 2-min denaturation at 94°C and followed by a 5-min final extension at 72°C. Thermal profile for GAPDH amplification: 30 cycles starting with denaturation of 1 min at 94°C, followed by 30 s of annealing at 54°C, 40 s of extension at 72°C. Thermal profile for *β*-actin amplification: 30 cycles starting with denaturation 1.5 min at 94°C, followed by 30 s at 60°C, and 1 min of extension at 72°C. Thermal profile for BSP amplification: 30 cycles starting with denaturation 1 min at 94°C, followed by 30 s of annealing at 55°C, and 40 s of extension at 72°C. Thermal profile for ErbB2 amplification: 30 cycles starting with denaturation 30 s at 94°C, followed by 30 s of annealing at 54°C, 40 s of extension at 72°C.

Thermal profile for Osteocalcin amplification: 30 cycles starting with denaturation 30 s at 94°C, followed by 30 s of annealing at 55°C, 40 s of extension at 72°C.

All PCR products were analysed by radioactive Southern blot using specific labelled probes. Radioactivity was quantified with a STORM phosphor reader.


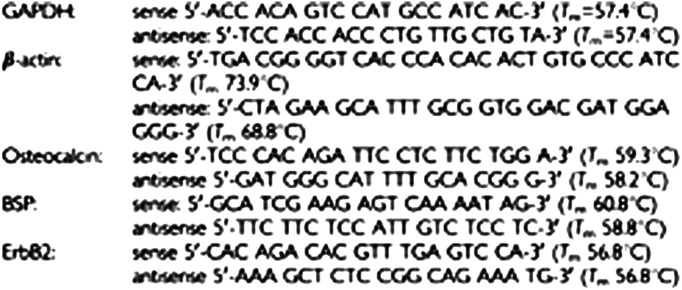


ErbB2 sense primer is located on exon 3; ErbB2 antisense primer is located on exon 5.

### Cell lines and cultures

U20S, SAOS II (available from ATCC catalogue), IOR/OS17, SARG, MOS, (obtained from Istituto Ortopedico Rizzoli di Bologna) were used as positive controls. U20S: human cell line from a differentiated osteosarcoma. SAOSII: human cell line from a femur osteosarcoma. IOR/OS17, SARG, MOS: human cell lines from osteoblastic osteosarcoma. All cell lines, were cultured in Dulbecco's modified essential medium (DMEM, GIBCO (Tulsa, OK, USA)) supplemented with 10% foetal bovine serum, 1% glutamine, penicillin streptomycin fungizon (PFS). All cell lines were grown at 37°C, in a CO_2_ 5% atmosphere.

## RESULTS

### Osteocalcin and Met are expressed at comparable levels in aphereses of patients and healthy stem cell donors

To detect tumour cells in aphereses of patients affected by metastatic osteosarcoma, we investigated the expression of the genes encoding for Osteocalcin and Met. Osteocalcin is a bone-specific protein localised in the extracellular matrix; Met, the tyrosine kinase receptor for HGF, is not expressed in osteoblasts, but is ectopically expressed in 60% of osteosarcomas.

**Figure 1:**
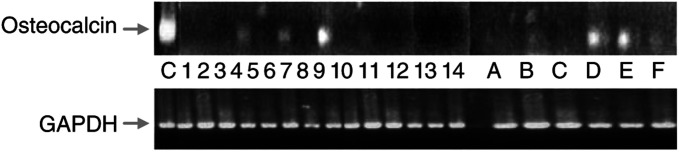
RT–PCR performed with osteocalcin-specific (upper panel) and GAPDH-specific (lower panel) primers on cDNAs obtained from 14 patients affected by metastatic osteosarcoma and six healthy haemopoietic stem cell donors. C: MG63 osteosarcoma cell line; 1–14: patient cDNAs; A–F: stem cell donors cDNAs. This and all the following experiments were done at least three times.

Using semiquantitative RT–PCR, we analysed Osteocalcin and Met expression in aphereses of healthy stem cell donors and patients. As shown in Figure 1

, Osteocalcin expression is elevated both in osteosarcoma patients and in healthy haemopoietic stem cell donors, without any significant difference between the two groups. The same results were obtained from the analysis of Met expression (data not shown). These data show that neither Osteocalcin nor Met are useful markers for the detection of tumour contamination.

### Average BSP expression is increased in patients affected by metastatic osteosarcoma

**Figure 2:**
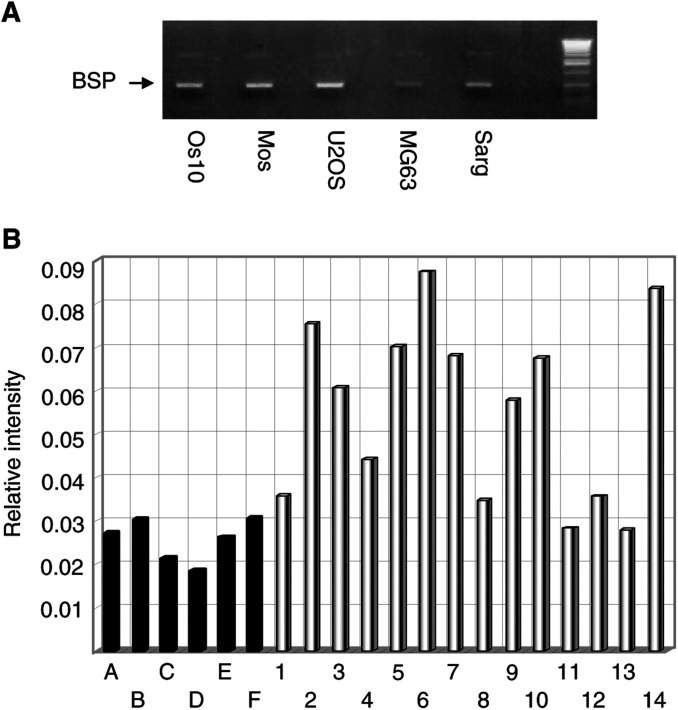
(**A**) cDNA amplification from human osteosarcoma cell lines with oligonucleotides specific for BSP. The size of the amplified fragment is 534 b. Os10, Mos, U2OS, MG63, Sarg: human osteosarcoma cell lines. (**B**) Schematic representation of the ratio between BSP and GAPDH expression in stem cell donors and in patients (for experimental details see the Materials and Methods section). A–F: stem cell donors; 1–14: patients affected by metastatic osteosarcoma.

BSP is an extracellular bone matrix protein that is mainly expressed by osteoblasts. It has also been shown that its expression is increased in prostate carcinomas where it is strongly associated with poor prognosis (Waltregny *et al*, 1998). We first showed that BSP mRNA is expressed in human osteosarcoma cell lines (Figure 2A

). On this premise, we compared BSP expression in aphereses of patients and of healthy haemopoietic stem cell donors by RT–PCR, followed by Southern blot. While control mRNA (GAPDH) levels are comparable, there is a significant difference in average BSP expression between the two groups. To measure the differences observed in Southern blot analysis, we quantified radioactivity of the bands by STORM phosphor reader and calculated the ratio between BSP and GAPDH values. Average BSP levels are 2.14 times higher in patients *vs* healthy donors. Moreover, 85% of patients (12 out of 14) display significantly higher BSP levels than all healthy donors (Figure 2B).

### ErbB2 is a useful marker for detection of tumour contamination in patients with ErbB2 expressing osteosarcomas

**Figure 3:**
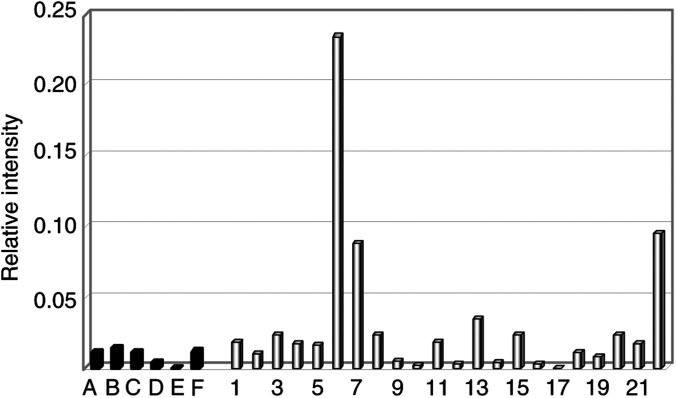
Schematic representation of the ratio between ErbB2 and *β*-actin expression in stem cell donors and in patients. A–F: stem cell donors; 1–22: patients affected by metastastic osteosarcoma. Patient numbering is the same as in Figure 2B.

The fourth marker we analysed is ErbB2. The ErbB2 gene, encoding for a tyrosine kinase of the EGF receptor family, is overexpressed in about 30% of human osteosarcomas and its expression correlates with a poor prognosis. We measured the levels of ErbB2 expression in patients and in healthy stem cell donors by RT–PCR followed by Southern blot. While levels of *β*-actin are comparable in patients and in donors, there is a remarkable difference in ErbB2 expression between the two groups. To quantify these differences, we measured radioactivity of the amplified bands and, for each sample, we calculated the ratio between ErbB2 and a control mRNA (*β*-actin). In all, 18% (four out of 22) of patients displayed levels higher than all controls. Positive patients showed values from seven to 16 times higher than healthy donors (Figure 3

).

**Figure 4:**
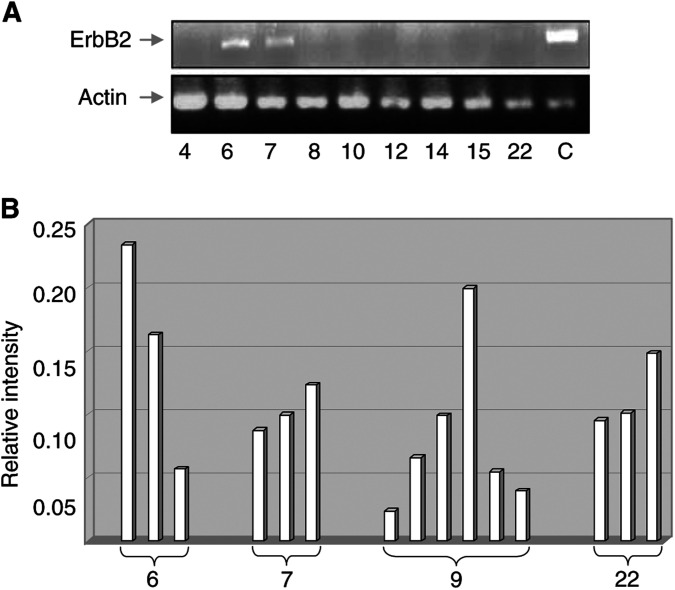
(**A**) RT–PCR performed with ErbB2-specific (upper panel) and *β*-actin-specific (lower panel) oligonucleotides. cDNAs were obtained from metastasis of nine patients. C: cDNA from MG63 osteosarcoma cell line. Patient numbering is the same as in Figure 3. (**B**) Schematic representation of the ratio between ErbB2 and *β*-actin expression in different aphaereses derived from ErbB2+ patients (patient numbering corresponds to Figure 3).

To demonstrate that ErbB2 expression in aphereses may be because of the presence of metastatic cells, we evaluated ErbB2 expression in lung metastasis derived from patients displaying high levels of ErbB2 in aphereses. As shown in Figure 4

, ErbB2 expression was evident in neoplastic tissues derived from these patients (samples 6, 7 and 22), but not in those derived from patients whose aphereses were negative.

Moreover, to evaluate if consecutive aphereses derived from the same patient display the same level of tumour contamination, we performed analysis of expression of ErbB2. As shown in Figure 4B, patients ErbB2+ displayed different values of ErbB2 in different aphereses. This observation suggests that the choice of the apheretic collection for reinfusion could decrease the risk of metastatic contamination.

## DISCUSSION

At present, treatment of high-grade metastatic osteosarcoma is unsatisfactory as 5 years overall survival is only 20–25%. Increase of dose intensity of chemotherapeutic agents (high-dose chemotherapy) may improve these poor results, but it is associated with an increase of haematological toxicity. Recent use of haemopoietic growth factors has allowed the reduction of neutropenia and has given the possibility of mobilising and harvesting an adequate number of haemopoietic CD34+ stem cells (Fagioli *et al*, 2002). Purified CD34+ stem cells, once reinfused after myeloablative treatment, can induce a faster haemopoietic recovery. However, reinfusion of stem cells has generated concern about the possibility of rescuing neoplastic cells contaminating the aphereses. Even if it is not yet known whether relapses are because of endogenous or reinfused cells, it is certainly important to investigate tumour contamination in leukaphereses.

Our objective was to find markers able to detect osteoblastic osteosarcoma contamination in aphereses of patients with metastatic osteosarcoma. Since, as yet, no specific antigen has been found for osteosarcoma, in order to reveal the presence of metastatic osteosarcoma cells, we looked for cells expressing either bone-specific markers or oncogenes that have been shown to play a role in osteosarcoma development. Among the markers belonging to the first group we analysed BSP and Osteocalcin; among the oncogenes described to play a role in osteosarcoma progression, we evaluated Met and ErbB2.

Our data indicate that neither Met nor Osteocalcin are useful markers to detect metastatic cells since they are expressed at comparable levels in aphereses of patients with metastatic osteosarcoma as well as those of healthy stem cell donors.

We obtained more interesting results with BSP, a protein produced by osteoblasts, whose expression is increased in osteosarcoma patients; nevertheless, quantitative difference between the two groups is not wide enough to use this molecule as a single marker in the detection of peripheral micrometastasis. On the contrary, since this molecule is expressed in the vast majority of primary osteosarcomas, its additional evaluation might be helpful for further characterization of patients.

The last molecule we considered for the detection of tumour contamination is ErbB2, a tyrosine kinase receptor of the EGF receptor family. Even if this is not a tumour-specific marker, it is overexpressed in 30% of osteosarcomas and it is associated with poor prognosis (Gorlick *et al*, 1999). In our experiments, we found that 18% of aphereses derived from patients affected by osteosarcoma were significantly positive for ErbB2 expression; lung metastasis derived from these same patients were also positive for ErbB2 expression. It is also important to point out that positivity of the apheresis was detected only in patients whose neoplastic tissues express ErbB2.

We can thus conclude that, although finding low ErbB2 levels does not exclude neoplastic bone marrow contamination, high levels of ErbB2 expression in aphereses might be evocative of micrometastasis. Another conclusion that can be drawn by this work is that aphereses obtained by the same patient can display a different degree of ErbB2 expression. It might be thus critical, when possible, to analyse the level of expression of ErbB2, to avoid reinfusion of blood cells contaminated by ErbB2+ cells. Moreover, if reinfusion of these positive aphereses is required, the possibility of purging them by use of anti-ErbB2 antibodies should be considered.

ErbB2 has been extensively studied for its involvement in different human tumours, such as breast cancer. Since also in these cases overexpression of this gene correlates with a poor prognosis, therapeutic approaches have been pursued to either decrease ErbB2 expression or functionally inactivate it. In fact, few years ago a monoclonal anti-ErbB2 antibody (Herceptin) became available and was used to treat patients with metastatic breast cancer; these trials showed a significant improvement of patient's outcome following this treatment (Piccart and Awada, 2000; Stebbing *et al*, 2000). If our data will be confirmed on larger numbers, it can be hypothesized that a similar application of Herceptin in ErbB2 overexpressing osteosarcomas could also result in treatment improvement. Moreover, many other therapeutical approaches targeting this molecule are under development, such as the use of monoclonal anti-ErbB2 antibodies conjugated with cytotoxic drugs (Azemar *et al*, 2000) single-chain antibodies, and DNA vaccines (Wei *et al*, 1999).
